# P11 Evaluation of *in vitro* activity of double β-lactam therapy and relationship with PBP activity in *Escherichia coli* isolates

**DOI:** 10.1093/jacamr/dlac053.011

**Published:** 2022-05-31

**Authors:** J Suich, M Van der Woude, D Wearmouth, P Burns, G Barlow

**Affiliations:** 1 Hull University Teaching Hospitals NHS Trust, Hull, UK; 2 University of York, York, UK

## Abstract

**Background:**

PBPs are involved in the construction of peptidoglycan, which is the major constituent of bacterial cell walls and the target of β-lactam antibiotics. There is little published research analysing the relationship between β-lactams with differing bacterial PBP targets and how they can be manipulated in combinations with respect to clinical or microbiological (e.g. resistance) outcomes (i.e. does expanded PBP activity via a combination lead to better *in vitro*/*in vivo* outcomes).

**Objectives:**

To systematically explore the relationship between double β-lactam therapy (with and without at least one partner being a β-lactamase inhibitor antibiotic such as co-amoxiclav) and *in vitro* activity against susceptible *Escherichia coli* strains.

**Methods:**

We systematically explored the relationship between double β-lactam therapy combinations against seven *E. coli* strains of variable resistance *in vitro*. This included fully susceptible isolates, ESBL producers and carbapenemase producers (CPEs). For each of 10 antibiotics, the MIC was determined individually, and subsequently in combination with 9 further antibiotics, using the MTS^™^ ‘cross’ synergy method (Liofilchem, 2012).

**Results:**

Overall, 86/630 (13.6%) of all combinations tested showed synergy and 408/630 (64.8%) were additive; 136/630 (21.6%) combinations showed indifference. Of the 86 ‘bug–drug’ combinations that showed synergy, 42/86 (49%) included ceftazidime/avibactam, representing 42/126 (33%) of all ceftazidime/avibactam-based combinations tested, and 56/86 (65%) of synergistic combinations covered PBP2. Synergy was most commonly detected in ESBL producers (58/86; 67% of combinations) and less frequently seen in CPEs (2/86; 2% of combinations) and fully susceptible isolates (8/86; 9% of combinations). Additive effects were seen in 92/180 (51%) combinations versus ESBLs, compared with 18/90 (20%) in CPEs, versus 154/180 (86%) in fully susceptible isolates. No antagonism was identified with any antibiotic combination.

**Conclusions:**

In the combinations tested, synergy or additive effects were common (78%); similar to our previous work with fosfomycin/β-lactam combinations (89%), but higher than we found with fosfomycin/non-β-lactam combinations (28%). Many of the synergistic bug–drug combinations identified contained a β-lactam inhibitor as a partner and/or provided PBP2 activity. This provisionally suggests a role for PBP2 (also targeted by avibactam) in synergy, although the presence of a β-lactamase inhibitor may also be important. Confirmation using an alternative method and mechanistic elucidation is required. The clinical/microbiological importance of such effects remains unclear.

**Figure 1.**
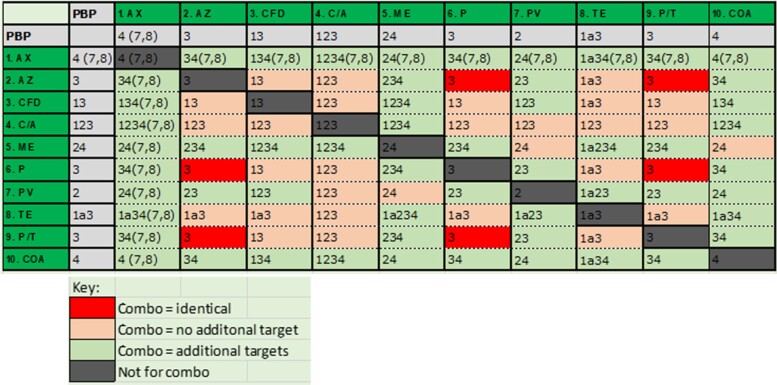
Conceptual matrix of antibiotics and the associated PBPs covered as monotherapy and combination therapy. 1, amoxicillin Etest; 2, aztreonam Etest; 3, ceftazidime Etest; 4, ceftazidime/avibactam Etest; 5, meropenem Etest; 6, piperacillin Etest; 7, (piv)mecillinam Etest; 8, temocillin Etest; 9, piperacillin/tazobactam Etest; 10, co-amoxiclav Etest.

